# Case report: Predictability of clinical response and rejection risk after immune checkpoint inhibition in liver transplantation

**DOI:** 10.3389/frtra.2023.1211916

**Published:** 2023-08-14

**Authors:** Jordi Yang Zhou, Dominik Eder, Florian Weber, Philipp Heumann, Katharina Kronenberg, Jens M. Werner, Edward K. Geissler, Hans J. Schlitt, James A. Hutchinson, Florian Bitterer

**Affiliations:** ^1^ Department of Surgery, University Hospital Regensburg, Regensburg, Germany; ^2^ Leibniz Institute for Immunotherapy, University Hospital Regensburg, Regensburg, Germany; ^3^ Institute for Pathology, University of Regensburg, Regensburg, Germany; ^4^ Department for Internal Medicine I, University Hospital Regensburg, Regensburg, Germany

**Keywords:** immune checkpoint inhibition, liver transplantation, hepatocellular carcinoma, immune monitoring, biomarker, clinical response, rejection risk

## Abstract

**Background:**

The approval of Atezolizumab / Bevacizumab therapy (Atezo/Bev) in 2020 opened up a promising new treatment option for patients with end-stage hepatocellular carcinoma (HCC). However, liver transplant (LTx) patients with HCC are still denied this therapy owing to concerns about ICI-induced organ rejection and lack of regulatory approval.

**Methods:**

A prospective observational study at a tertiary liver transplant centre monitored the compassionate, off-label use of Atezo/Bev in a single, stable LTx recipient with non-resectable HCC recurrence. Close clinical, laboratory and immunological monitoring of the patient was performed throughout a four-cycle Atezo/Bev treatment. Measured parameters were selected after a systematic review of the literature on predictive markers for clinical response and risk of graft rejection caused by ICI therapy.

**Results:**

19 articles describing 20 unique predictive biomarkers were identified. The most promising negative prognostic factors were the baseline values and dynamic course of IL-6, alpha-fetoprotein (AFP) and the AFP/CRP ratio. The frequency of regulatory T cells (Treg) reportedly correlates with the success of ICI therapy. PD-L1 and CD28 expression level with the allograft, peripheral blood CD4^+^ T cell numbers and Torque Teno Virus (TTV) titre may predict risk of LTx rejection following ICI therapy. No relevant side effects or acute rejection occurred during Atezo/Bev therapy; however, treatment did not prevent tumor progression. Absence of PD-L1 expression in pre-treatment liver biopsies, as well as a progressive downregulation of CD28 expression by CD4^+^ T cells during therapy, correctly predicted absence of rejection. Furthermore, increased IL-6 and AFP levels after starting therapy, as well as a reduction in blood Treg frequency, correctly anticipated a lack of therapeutic response.

**Conclusion:**

Atezo/Bev therapy for unresectable HCC in stable LTx patients remains a controversial strategy because it carries a high-risk of rejection and therapeutic response rates are poorly defined. Although previously described biomarkers of rejection risk and therapeutic response agreed with clinical outcomes in the described case, these immunological parameters are difficult to reliably interpret. Clearly, there is an important unmet need for standardized assays and clinically validated cut-offs before we use these biomarkers to guide treatment decisions for our patients.

## 1. Introduction

Hepatocellular carcinoma (HCC) represents the third leading cause of cancer deaths worldwide (1). Therapeutic options for advanced HCC that cannot be treated curatively by surgical resection, liver transplantation or radiofrequency ablation are limited. In end-stage cases, apart from transarterial chemoembolisation and radioembolisation, available drug therapies include the multikinase inhibitors (MKI) sorafenib and lenvatinib for first-line treatment, cabozantinib and regorafenib for second-line treatment after progression on sorafenib; and the anti-angiogenesis drug ramucirumab as second-line after progression on sorafenib. Beyond its limited oncological effectiveness, use of MKI therapy has been restricted by its pronounced side effects (2). Consequently, advanced HCC still carries a poor prognosis and represents a significant unmet medical need.

Approval of cancer immunotherapies, especially immune checkpoint inhibitor (ICI) therapy, has opened new clinical perspectives for cancer patients and is fast becoming one of the main pillars of cancer treatment. Early studies in advanced HCC suggested that only 15%–20% of patients benefited from anti-PD-1 monotherapy, including Checkmate040 (NCT01658878) (3, 4) and KEYNOTE-224 (NCT02702414) (5, 6). More recently, combination therapy using atezolizumab, an anti-PD-L1 immune checkpoint inhibitor, plus bevacizumab, an anti-VEGF neoangiogenesis inhibitor, showed promising results in the randomized phase 3 IMbrave150 trial (NCT03434379). Compared to sorafenib, advanced HCC patients had superior overall survival (OS) (19.2 months vs. 13.4 months, HR = 0.66, 95% CI = 0.52–0.85, *P* = 0.0009) and progression-free survival (PFS) (6.9 months vs. 4.3 months, HR = 0.65, 95% CI = 0.53–0.81, *P* = 0.0001) (7). In 2020, the United States Food and Drug Administration (FDA) and the European Medicines Agency (EMA) approved Tecentriq (Atezolizumab) in combination with Avastin (Bevacizumab) as the new standard of care for patients with unresectable or metastatic HCC who have not received prior systemic therapy. Critically, the approved indication excludes liver transplant (LTx) patients owing to the potential risk of acute graft rejection.

Immune-related adverse events (irAEs) are the result of dysregulated immune activation following ICI therapy. Checkmate040 and KEYNOTE-224 indicated that around 25% of advanced HCC patients develop grade 3 or 4 irAEs, including severe or life-threatening pneumonitis, colitis, hepatitis, dermatitis, arthritis, encephalitis and other complications (3–6). With the goal of sparing unnecessary toxicity and comorbidity, as well as offering therapeutic alternatives to LTx patients with recurrent HCC, efforts have been made to identify biomarkers that predict therapeutic responses and risk of irAEs (8–10). Unfortunately, success has been limited despite the characterization of novel mechanisms for ICI-induced hepatitis. In particular, in a cohort of melanoma patients treated with a combination of anti-PD-1 and anti-CTLA-4, patients with a chronic or recurrent immune response against cytomegalovirus (CMV) showed an expansion of effector memory CD4^+^ T cells that predisposed to hepatitis (11). However, it is not presently clear whether such biomarkers and preventative strategies can be directly translated to liver transplant recipients with unresectable recurrent HCC. In a comprehensive review of 28 cases of ICI therapy in LTx heavily pre-treated patients, mostly HCC recurrent patients, graft rejection was observed in 32% of the cases and only 25% responded to therapy (12). Based upon the limited number of reported ICI-treated HCC cases in LTx recipients, it is hard for clinicians to assess the potential risks and benefits of off-label treatment in individual cases; therefore, we urgently need reliable clinical markers to guide effective treatment decisions (13).

## 2. Case description

### 2.1. History of recurrent HCC after liver transplantation

**Figure 1:**
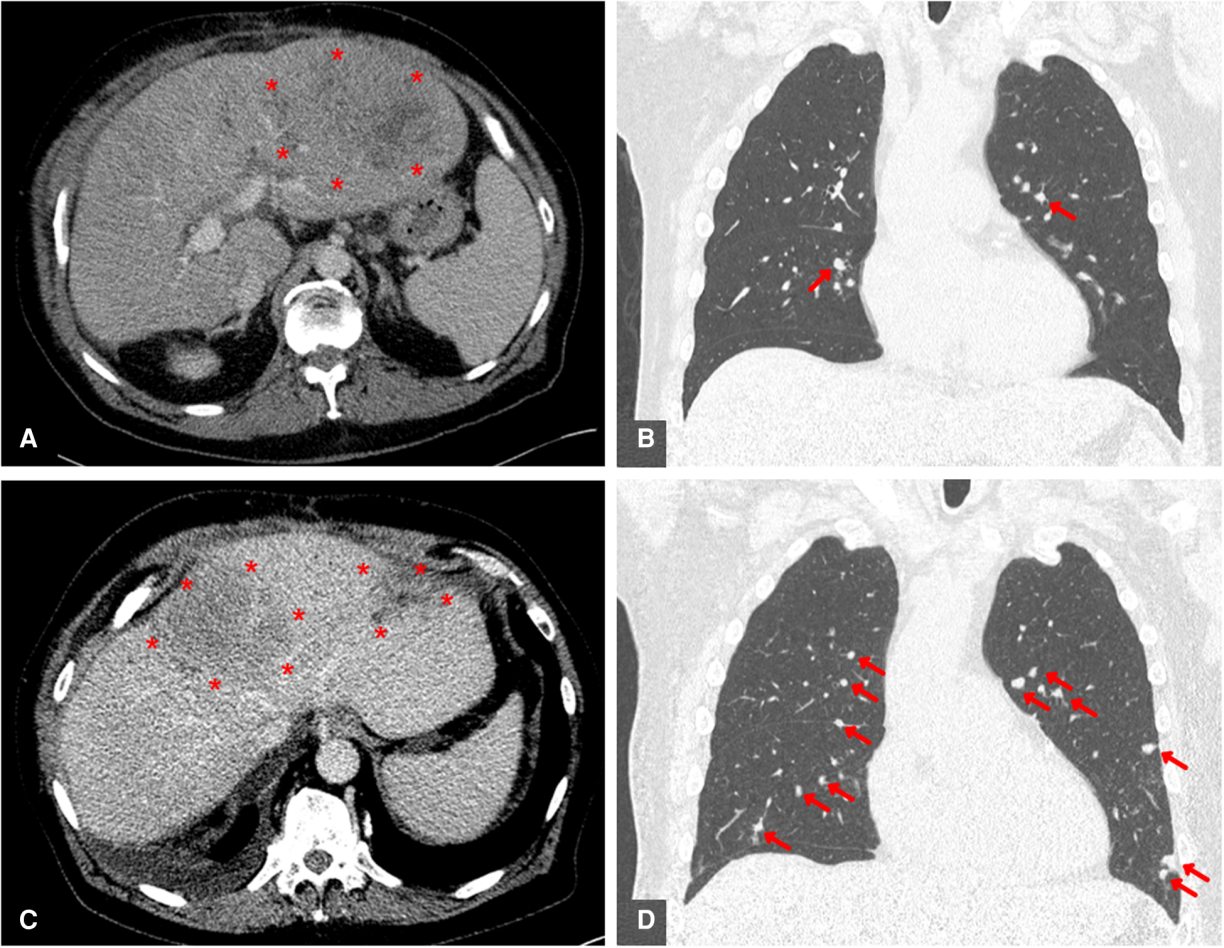
Contrast-enhanced CT scans before initiation of ICI therapy and as interim examination after 3 cycles of atezolizumab / bevacizumab treatment. Both intrahepatic (A + C) and intrapulmonary (B + D) HCC metastases are marked in colour. (**A**) Pretherapeutic axially reconstructed abdominal CT scan showing a large recurrence of HCC in the left liver lobe. (**B**) Pretherapeutic coronary reconstructed thoracic CT scan showing a pulmonary metastasis of HCC. (**C**) Axially reconstucted abdominal interim CT scan showing an example of a pronounced progression of intrahepatic HCC. (**D**) Coronary reconstucted thoracic interim CT scan showing marked progression of pulmonal HCC metastases.

A 62-year-old man presented in 2021 to our liver transplant outpatient clinic with a first HCC recurrence in liver segment IVa/VIII. The patient was the recipient of an orthotopic liver transplant in 2010, necessitated by alcohol-related liver cirrhosis with intrahepatic, multilocular HCC. At the time of recurrence in the graft, radiofrequency ablation was started with curative intent, but was ineffective. At short-term re-staging, several new intrahepatic HCC foci were identified, as well as new pulmonary metastases (Figures 1A,B). The presence of extrahepatic metastases meant that therapies limited to the liver, such as TACE, SIRT or multiple ablations, were inappropriate. With no other potentially curative options, the transplanted patient was considered for off-label treatment with Atezo/Bev therapy as an alternative to the guideline-recommended “standard of care” with an MKI.

An extensive risk assessment was undertaken, including clinical and laboratory investigations. The patient's general clinical condition was normal for age, with an ECOG status of 0 and Karnofsky index of 100%. We further excluded comorbidities as contraindications for immunotherapy, including synchronous tumors, autoimmune diseases, and occult infections (Supplementary Figure 2). Liver transplant function was stable under triple immunosuppression with Sirolimus (target trough levels of 3–5 µg/L) and mycophenolate mofetil (1,000 mg twice a day). Balancing the potential oncological success of ICI therapy against its risk of causing rejection, we added low-dose prednisolone (2.5 mg once daily), knowing that it may be associated with reduced tumor response (14). No clinically validated investigations provided an estimate of rejection risk; therefore, the patient was offered Atezo/Bev therapy with a 32% attendant risk of transplant failure and 25% therapy response rate (12). The patient provided full, informed consent to treatment.

**Figure 2:**
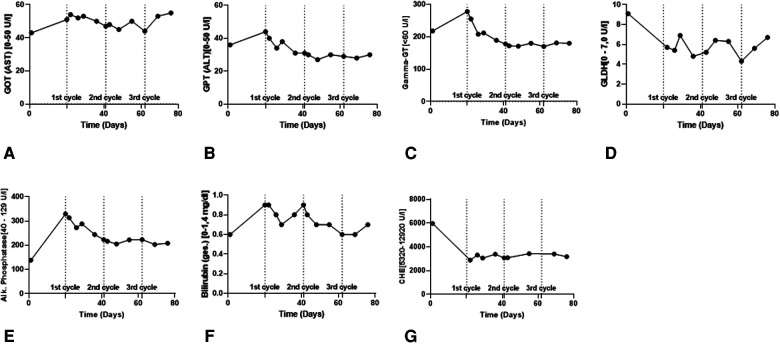
Routine biochemical liver parameters in the course of ICI therapy. (**A,B**) Liver enzymes (GOT & GPT) to assess the vitality of the hepatocytes. (**C–E**) Cholestasis parameters (*γ*GT, GLDH & AP) to assess the vitality of the cholangiocytes. (**F,G**) Bilirubin and CHE for assessing metabolic functionality of the transplanted liver.

Four cycles of 1,200 mg Atezolizumab plus 15 mg/kg Bevacizumab were administered at three-week intervals. The patient was kept under close surveillance for signs of acute transplant rejection. In the first week after Atezo/Bev infusion, we reviewed the patient at least every second day; during breaks in therapy, he was seen at least once per week. The patient never developed clinical or biochemical signs of a serious irAE. Over time, the patient complained of mild symptoms, including moderate fatigue, mild diarrhea, and sleeping problems. Transplant function remained stable throughout and following Atezo/Bev therapy. Specifically, liver enzyme values were stable after each cycle (Figure 2) and ultrasound imaging revealed no signs of acute liver rejection. Unfortunately, interim CT scans revealed further HCC progression with multiple intrahepatic foci and new pulmonary metastases (Figures 1C,D). Hence, despite completing a full course of Atezo/Bev therapy without a serious irAE or acute rejection occurring, the treatment was ultimately ineffective.

This case illustrates the difficult clinical decision of whether to offer immunotherapy to liver transplant recipients with recurrent HCC. At the present time, neither the risk of irAE or rejection, nor the likelihood of clinical response can be accurately predicted using clinically validated tests. Therefore, we next asked what experimental methods could be of use.

### 2.2. Hepatic PD-L1 expression as a marker of rejection risk

Programmed death-ligand 1 (PD-L1) is a cell-surface ligand widely expressed on tumor cells. Binding of Atezolizumab to PD-L1 blocks its interaction with PD-1, an inhibitory receptor expressed by T cells, which releases them from functional inhibition by tumors. In HCC, PD-L1 is expressed by approximately 10%–20% of tumor cells and higher levels are generally associated with tumor aggressiveness and poor survival (15). A phase I trial of Atezo/Bev therapy in HCC patients concluded that high expression of PD-L1 within tumors correlated with better responses and longer progression-free survival (PFS) times (16).

In many experimental models of transplant acceptance, disrupting PD-L1 interactions leads to acute allograft rejection (17). Likewise, transcriptomic profiling of tolerated and stably accepted liver transplants has revealed the importance of PD-L1 in controlling T cell alloimmunity in patients (18). In the context of ICI after liver transplantation, a retrospective study investigated intragraft PD-L1 expression as a marker of acute transplant rejection risk in liver-transplanted patients with recurrent HCC who underwent anti-PD-1 therapy (19). PD-L1 expression by hepatocytes was associated with a greater risk of acute rejection. Similarly, a prospective single-arm study of anti-PD-1 for recurrent malignancy after liver transplantation found that 5 patients lacking PD-L1 expression in their grafts remained stable during therapy, whereas a single PD-L1^+^ patient underwent acute rejection (20). Given the limited available data, hepatic PD-L1 expression is an interesting, but unvalidated biomarker of rejection risk.

**Figure 3:**
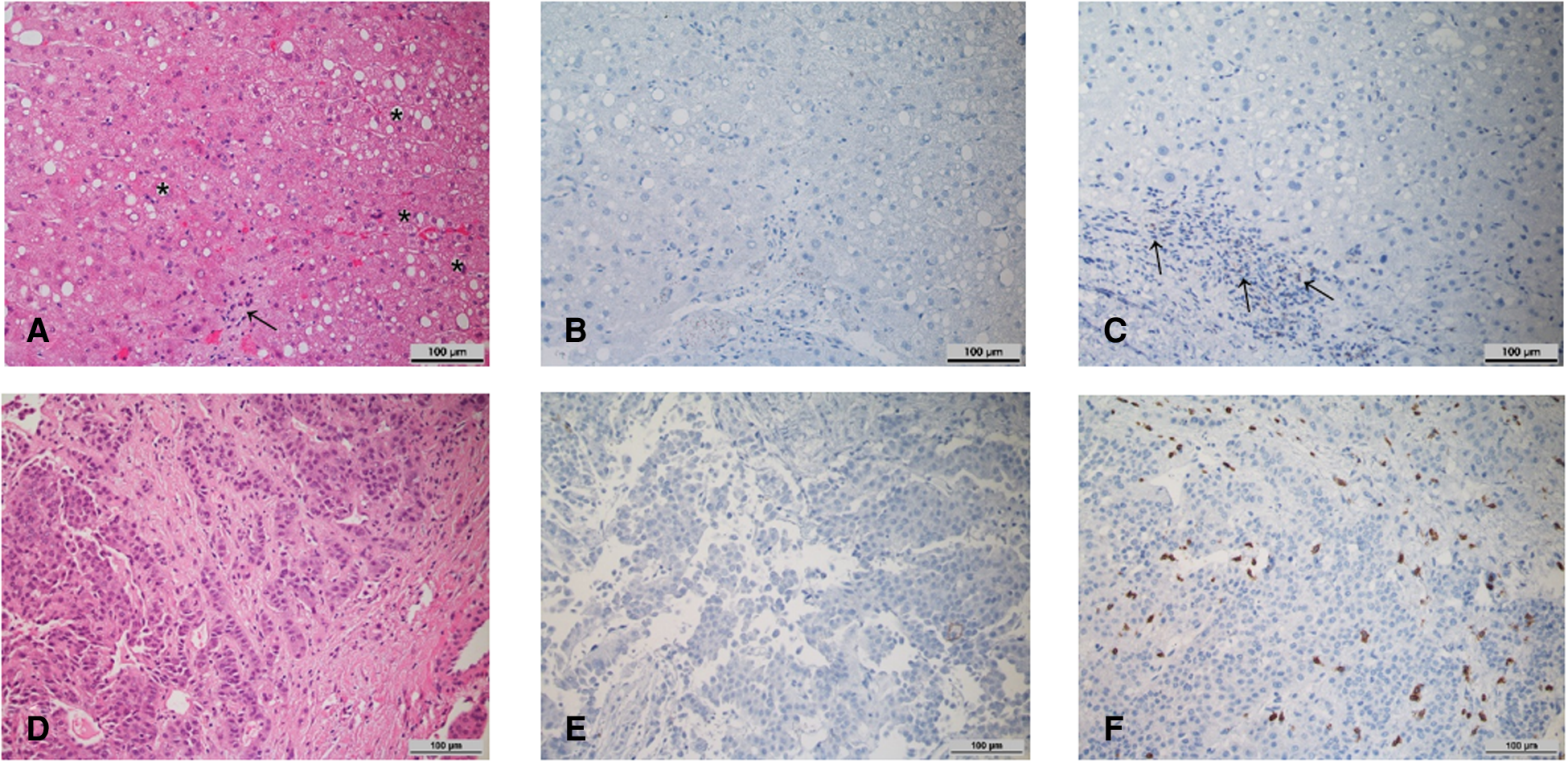
Histopathology of biopsies of the transplant liver (**A–C**) as well as intrahepatic HCC foci (**D,E**) prior to initiation of immune checkpoint therapy (200x magnification). (**A**) HE-stained representative section of the transplant liver biopsy. * intrahepatic fat vacuoles, → inflammatory infiltrate. (**B**) Corresponding immunohistochemical staining for PD-L1. (**C**) Corresponding immunohistochemical staining for PD-1. → inflammatory infiltrate with scattered PD-1 positive lymphocytes. (**D**) HE-stained representative section of HCC-infiltrated liver biopsy. (**E**) Corresponding immunohistochemical staining for PD-L1. (**F**) Immunohistochemical staining for CD3.

Histopathological examination of a liver biopsy taken before starting Atezo/Bev therapy revealed acute steatohepatitis with distinct fatty degeneration of the hepatocytes (70%) and fibrosis (Ishak fibrosis grade 3–4) (Figure 3A). There were no signs of rejection. A biopsy from the tumor showed solid and trabecular infiltrates of malignant and moderately-differentiated hepatocellular carcinoma (G2) with typical shift of the nuclear-plasma relation, small-foci necrosis and marked fragmentation (Figure 3D). Immunohistochemical staining of tumor-free liver specimens for PD-L1 and PD-1 was completely negative in hepatocytes. The intrahepatic immune cells, especially the lymphocytes, showed minimal expression of PD-L1 (< 1%) and about 5% positivity for PD-1 (Figures 3B–C). Immunohistochemistry from the tumor biopsy indicated a Tumor Proportion Score (TPS) of 0%, an Immune Cell Score (ICS) <1% and a Combined Positive Score (CPS) <1% (Figure 3E), registering a scattered CD3-positive intra/peritumoral T lymphocyte infiltrate (Figure 3F). Thus, we add another case of a stable LTx patient lacking histological PD-L1 expression that safely underwent ICI therapy without rejection.

The question of whether to start of anti-PD-L1 treatment knowing there is minimal PD-L1 expression (<1%) is important. Unfortunately, we cannot conclusively answer whether treatment failure was predictable in this case. In contrast to other tumor entities such as melanoma, HNSCC or urothelial carcinoma, where intratumoral expression of PD-L1 or PD-1 has been shown to be predictive, this is not consistent in HCC. For example, in Checkmate040, response rates were comparable across all subgroups (PD-L1 < or ≥1%) (3, 4). On the other hand, KEYNOTE-224 correlated ICI response with PD-L1 expression under certain conditions (5, 6). All of these limitations of using PD-L1 expression as a stand-alone biomarker are reflected in the FDA's deliberate disregard of PD-L1 expression for ICI approval in the treatment of HCC (21).

### 2.3. Plasma markers of clinical response

**Figure 4:**
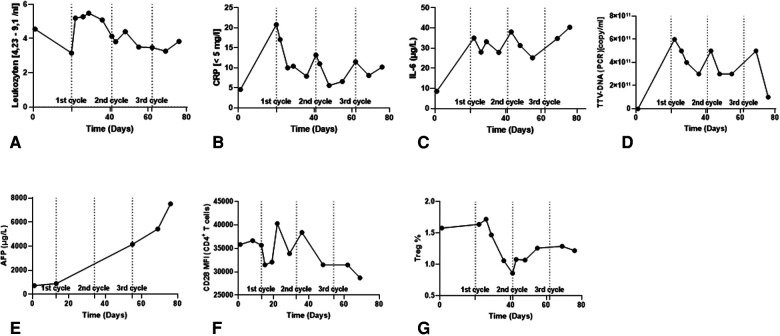
Immunomonitoring data in the course of ICI therapy. (**A–C**) Clinical routine Inflammatory parameters. (**D**) Torque Teno Virus level (TTV) as a surrogate parameter for the patient's immunocompetence. (**E**) AFP tumor marker level. (**F**) CD28 expression level in CD4^+^ T cells in peripheral blood. (**G**) Frequency of Tregs in the peripheral blood.

IL-6 has been proposed as a predictor of therapeutic response in patients receiving Atezo/Bev therapy (22). In a recent study, patients with elevated baseline IL-6 levels (>4.77 pg/ml) were significantly disposed to shorter PFS. IL-6 mediates inflammation and promotes T cell infiltration of tumors. On the other hand, in some cancers, including HCC, IL-6 can suppress tumor immunity by recruiting myeloid-derived suppressor cells (MDSCs) (22). When IL-6 levels were measured in our patient, they exceeded the cut-off associated with a positive clinical responses. We observed a persistent ∼4-fold increase in IL-6 levels after starting immunotherapy (Figure 4C).

Presently, alpha-fetoprotein (AFP) is widely applied as a non-invasive monitoring biomarker of HCC tumor burden and aggressiveness. AFP ≥ 400 ng/ml is a negative prognostic factor for overall survival (OS) and is used as a stratification factor for unresectable HCC patients in clinical trials. In the context of Atezo/Bev therapy, AFP might be a useful marker for monitoring clinical effectiveness. It was recently reported that a ≥75% decrease or ≤10% increase in AFP levels from baseline to 6 weeks has been associated with improved OS and PFS (23). Our patient presented with an AFP of 731 ng/ml at baseline, and 6 weeks after the start of therapy there was a progressive increase in AFP levels by ∼7.5-fold (5,435 ng/ml) (Figure 4E).

Combining AFP and C-Reactive Protein (CRP) serum levels has been suggested as an scoring system to predict clinical responses and survival in advanced HCC patients treated with Atezo/Bev therapy (24, 25). Lower scores are associated with better PFS and OS. A maximum score of 2 is assigned to patients with baseline AFP ≥ 100 ng/ml and CRP ≥ 1 mg/dl. Based on this parameter, our patient had a score of 1 (AFP 731 ng/ml; CRP 0.46 mg/dl), indicative of inferior OS and inferior PFS (Figures 4B,E). In relation to our patient, the IL-6 and AFP/CRP scores lead to contradictory prognoses, suggesting that further aspects need to be considered.

### 2.4. Torque Teno Virus as a marker of rejection risk

Torque Teno Virus (TTV) load is widely used to monitor the intensity of general immunosuppression in transplant recipients. In healthy individuals, TTV load is controlled by T cell responses; therefore, adequately immunosuppressed transplant recipients have higher viral titers. A normalized TTV load in a transplant recipient suggests under-immunosuppression and an elevated risk of rejection (26). Our patient showed a consistent reduction in TTV load after each cycle of Atezo/Bev therapy, suggesting intensified T cell activity; however, TTV titers recovered quickly after each cycle (Figure 4D). TTV values should be interpreted with caution. In the context of graft tolerance, Atezo/Bev did not lead to a stable increase in TTV titers indicating adequate immunosuppression. As for the efficacy of the therapy, TTV load has not been established as an indicator of therapy responsiveness in LTx patients. In the absence of a validated normal TTV titer range in this particular constellation of ICI therapy plus low dose of prednisolone, it may be mechanistically interesting, but is currently unhelpful in guiding clinical decisions.

### 2.5. Flow cytometry markers of clinical response and rejection risk

A study analyzing samples from the G030140 phase 1b study found that higher numbers of regulatory T cells (Treg) in blood was associated with longer PFS in patients treated with Atezo/Bev therapy compared to atezolizumab alone. Furthermore, Treg frequencies tended to decline after immunotherapy. In our patient, baseline Treg frequency was 1.6% and we observed a reduction to 0.9% Tregs after starting therapy, which did not recover during follow-up (Figure 4G).

Acutely increased CD28 expression by peripheral blood CD4^+^ T cells has been reported as a predictive marker for risk of acute liver transplant rejection (27). Our flow cytometry results indicated a progressive downregulation in CD28 MFI during Atezo/Bev therapy, suggesting our patient did not have an elevated risk of rejection (Figure 4F).

## 3. Diagnostic assessment

### 3.1. Case study

We report the case of a single liver transplant recipient who presented to the liver transplant outpatient clinic at University Hospital Regensburg with recurrent HCC. According to German Pharmaceutical Law, the patient was treated off-label with atezolizumab plus bevacizumab as a compassionate-use case. The patient provided full, informed written consent to laboratory investigations and collection of case details for publication.

### 3.2. Systematic review protocol and data extraction

Selection of publications and data extraction were performed in a standardized manner. We searched Medline at the National Library of Medicine through the NCBI website on 20-OCT-2022. Our search terms were “immunotherapy”, “atezolizumab”, “bevacizumab”, “HCC”, “biomarkers”, “liver”, “response”, ‘acute cellular rejectioń and synonyms. We followed up on relevant citations from articles returned in our original search. In total, we found 19 articles describing 20 unique biomarkers (Supplementary Figure S1).

### 3.3. Clinical laboratory investigations

Before starting ICI therapy, tissue biopsies were taken under sonographic guidance from both the transplanted liver and intrahepatic foci of HCC recurrence. An in-house accredited pathology laboratory (Institute of Pathology, UKR) performed histopathological investigations. For immunohistochemical staining, PD-L1 was detected with antibody clone 22C3 (DAKO, Denmark), PD-1 was detected with NAT105 (abcam, UK) and CD3 was detected with #A0452 (DAKO, Denmark). Routine Biochemistry and Haematology were performed by an in-house accredited diagnostic laboratory (Institute of Clinical Chemistry and Laboratory Medicine, UKR). Blood samples for this purpose were part of routine clinical examinations that occurred several times a week.

### 3.4. Flow cytometry

Whole blood samples were collected in EDTA tubes by peripheral venipuncture and stored at 4 °C until processing began within 4 h. Samples were prepared for flow cytometry analysis according to previously published methods (11). Detailed step-by-step protocols are available through Protocol Exchange (28). Briefly, whole blood samples were stained with DURAClone IM antibody panels (DURAClone IM Count Tube, C00162; DURAClone IM phenotyping BASIC Tube, B53309; DURAClone IM B cells Tube, B53318; DURAClone IM Dendritic Cell Tube, B53351; DURAClone IM Granulocytes Tube, B88651; DURAClone IM T cell Subsets Tube, B53328; DURAClone IM TCRs Tube, B53340; DURAClone IM Treg Tube, B53346; all from Beckman Coulter, Germany). Data were recorded with a Navios™ cytometer running Cytometry List Mode Data Acquisition from Beckman Coulter. Analyses were performed using Kaluza version 2.1 (Supplementary Figure S3).

## 4. Discussion

Recurrent hepatocellular carcinoma is a significant problem after liver transplantation with few satisfactory treatment options. Immunotherapy for HCC in non-transplant patients is a promising approach, but its translation to liver transplant recipients raises critical questions about likely effectiveness and the risk of precipitating acute rejection. With sparse evidence, transplant clinicians are forced to make challenging decisions about using off-label immunotherapies in their patients. Achieving an optimal balance between immunosuppression and cancer immunotherapy is crucial to a successful outcome. Faced with this dilemma, we asked ourselves whether previously reported markers of clinical efficacy or rejection risk could help in decision-making. Of the parameters described in the literature, some, such as IL-6, AFP or CRP, are routinely measured in the clinic, while others require flow cytometry or invasive immunohistochemistry.

Although our measurements of parameters that predict rejection risk and clinical response broadly agreed with the observed clinical outcome in this case, we must be cautious about the general utility of these markers. Because of the non-validated discriminatory cut-off values of these markers, as well as the small numbers of reported patient outcomes, a final answer to the question regarding their prognostic value is not yet possible. The fact that the dynamic course of some parameters during ICI therapy, rather than baseline measurements, is important for prediction complicates decision-making.

In addition, other markers such as TGF-beta (28, 29) and certain microRNAs (30–32) have been described to have predictive potential for the success of ICI therapy. It remains unclear, to what extent these findings, which were all studied in patients without liver transplants, are applicable to transplant patients.

This clinical challenge of deciding about ICI therapy in transplant patients will become even more complex in the future as new agents or combinations besides Atezo/Bev gain regulatory approval for treatment of non-resectable HCC. Recently, the FDA approved of the combination of durvalumab, an anti PD-L1 antibody, and tremelimumab, an anti-CTLA-4 antibody, after the HIMALAYA Study showed an improvement in overall survival for patients with non-resectable HCC (33). Similarly the LEAP002 study (34, 35) demonstrated the benefit of pembrolizumab, a PD-1 inhibitor, used together with the TKI lenvatinib. Because of fast-paced advances in ICI therapy for HCC is fast, it is unclear whether a single predictive marker or marker combination is capable of predicting efficacy and rejection risk in all circumstances. All this underlines the importance of further clinical studies.

## Data Availability

The raw data supporting the conclusions of this article will be made available by the authors, without undue reservation.
