# Supplementary material for: Case report: Predictability of clinical response and rejection risk after immune checkpoint inhibition in liver transplantation
Source: Front Transplant. 2023 Aug 14;2:1211916. doi: 10.3389/frtra.2023.1211916 (PMC11235248; doi:10.3389/frtra.2023.1211916)
Supplement: Supplementary file 1 [file Datasheet1.pdf]

Biomarker	Author	Year	Type of study	Cancer type	Cohort size	Treatment	Timepoint	PMID	Response	Graft tolerance	Comment
NLR	Eso et al.	2021	Prospective	HCC	40	Atezo/Bev	Baseline	34677270	Yes	NA	Patients with disease control had a significantly lower NL ratio
% CD4+ CD154+ T cells	Boix et al.	2021	Prospective	NA	30	LTx	Baseline, 7d post-transplant & 14d post-transplant	33025622	NA	Yes	Significantly higher percentage in ACR
% CD8+ CD154+ T cells	Boix et al.	2021	Prospective	NA	30	LTx	Baseline, 7d post-transplant & 14d post-transplant	33025622	NA	Yes	Significantly lower percentage in ACR
HLA-DRB1 mismatch	Boix et al.	2021	Prospective	NA	30	LTx	Baseline	33025622	NA	Yes	Higher risk of ACR
CD28 expression in CD4+ T cells	Minguela et al.	1997	Prospective	NA	55	LTx	Baseline vs after transplant	9123102	NA	Yes	CD28 MFI increased in ACR patients
% IL-2+ CD3+ CD8+ T cells	Boleslawski et al.	2004	Prospective	NA	21	LTx	Baseline	15223897	NA	Yes	Higher risk of ACR
% IFNg+ CD8+ T cells	Millán et al.	2013	Prospective	NA	47	LTx	Baseline	23265966	NA	Yes	>55.8% identified as cut-off for high risk of ACR
% IL-17+ CD4+ T cells	Fan et al.	2012	Prospective	NA	76	LTx	Acute rejection	23232631	NA	Yes	Higher in ACR group vs NACR
AEC	Barnes et al.	2003	Prospective	NA	112	LTx	On the day or one day before biopsy	12694065	NA	Yes	Elevated AEC is a risk factor for ACR
Vδ1/ Vδ2 ratio	Zhao et al.	2013	Prospective	NA	34	LTx	After transplant	23222896	NA	Yes	Higher ratio in graft tolerated patients
Vδ1/ Vδ2 ratio	Martínez-Llordella et al.	2007	Prospective	NA	16	LTx	After transplant	17241111	NA	Yes	Higher numbers in tolerant recipients compared to non-tolerant patients or healthy individuals.
Vδ1/ Vδ2 ratio	Puig-Pey et al.	2010	Prospective	NA	314	LTx	After transplant	20477999	NA	Yes	Expansion of Vδ1 T cells is a common finding in transplant recipients
NLR	Tada et al.	2022	Retrospective	HCC	249	Atezo/Bev	Baseline	35170529	Yes	NA	Lower ratio had better prognosis
PD-L1+ in TILs	Cheng et al.	2022	Retrospective	HCC	135	Atezo/Bev	Prior to therapy	34902530	Yes	NA	≥1% associated with greater PFS & OS
T-effector signature	Zhu et al.	2022	Prospective	HCC	90	Atezo/Bev	Prior to therapy	35739268	Yes	NA	High expression of T effector signature (GZMB, PRF1, CXCL9) associated with clinical response and longer PFS
CD274	Zhu et al.	2022	Prospective	HCC	91	Atezo/Bev	Prior to therapy	35739268	Yes	NA	High expression associated with higher PFS & OS
Intratumoral CD8+ T cells	Zhu et al.	2022	Prospective	HCC	86	Atezo/Bev	Prior to therapy	35739268	Yes	NA	Higher density in IHC associated with higher PFS & OS
Treg signature	Zhu et al.	2020	Prospective	HCC	91	Atezo/Bev	Prior to therapy	NA	Yes	NA	Higher expression of Treg signature (CCR8, BATF, CTSC, TNFRSF4, FOXP3, TNFRSF18, IKZF2 & IL2RA) associated with longer PFS
PD-L1+ in tumor cells	Munker et al.	2018	Retrospective	HCC	5	LTx + anti-PD-1	After transplant	30228883	NA	Yes	Liver biopsies without evidence of PD-L1 expression are indicative of graft tolerance
PD-L1+ in tumor cells	Shi et al.	2021	Prospective	HCC	18	LTx + anti-PD-1	After transplant	32897657	NA	Yes	Liver biopsies without evidence of PD-L1 expression are indicative of graft tolerance
VEGFR expression	Zhu et al.	2020	Prospective	HCC	91	Atezo/Bev	Prior to therapy	NA	Yes	NA	Higher expression associated with longer PFS
Myeloid inflammation signature	Zhu et al.	2020	Prospective	HCC	91	Atezo/Bev	Prior to therapy	NA	Yes	NA	Higher expression associated with longer PFS
TREM1/MDSC signature	Zhu et al.	2020	Prospective	HCC	91	Atezo/Bev	Prior to therapy	NA	Yes	NA	Higher expression associated with longer PFS
AFP	Yavuz et al.	2021	Prospective	HCC	208	Atezo/Bev	6 weeks after treatment	34595140	Yes	NA	≥75% decrease or ≤10% increase associated with improved OS & PFS
AFP	Finn et al.	2020	Prospective	HCC	336	Atezo/Bev	Baseline	32402160	Yes	NA	AFP<400ng/ml associated with improved OS & PFS
PD-L1+ in tumor cells	Cheng et al.	2022	Retrospective	HCC	135	Atezo/Bev	Prior to therapy	34902530	Yes	NA	≥1% associated with greater PFS & OS
Anti-drug antibody	Galle et al.	2021	Prospective	HCC	336	Atezo/Bev	Baseline	NA	Yes	NA	ADA negative patients had improved OS vs sorafenib
IL-6	Myojin et al.	2022	Prospective	HCC	64	Atezo/Bev	Baseline	35205631	Yes	NA	IL-6 high group had shorter PFS & OS vs IL-6 low group

Supplementary Figure 1: Overview of the results of the systematic literature review to identify parameters for predicting treatment response and induction of graft rejection by ICI therapy in HCC recurrence after liver transplantation.

A

Routine laboratory parameters	pre-therapeutic baseline	Range
Leukocytes	4,55 /nl	4,23 – 9,1
Haemoglobin	12,7 g/dl	13,7 – 17,5
Platelets	78 /nl	163 - 337
Sodium	140 mmol/l	136 – 145
Potassium	4,0 mmol/l	3,4 – 4,5
Creatinine	1,35 mg/dl	0,67 – 1,17
GFR (CKD-EPI) Crea	54 ml/min/1,73 qm	
Urea	60 mg/dl	16,6 – 48,5
TSH (basal)	3,61 mIU/l	0,27 – 4,2
LDH	163 U/l	0 – 250
Lipase	83 U/l	13 – 60
CK	124 U/l	< 190
Ferritin	48,2 ng/ml	30 – 400
Transferrin	293 mg/dl	200 – 360
NT-proBNP	116 pg/ml	< 376

B

Graft liver function	pre-therapeutic baseline	Range
GOT (AST)	43 U/l	0 – 50
GPT (ALT)	36 U/l	0 – 50
GLDH	9.1 U/l	0 – 7,0
Gamma-GT	218 U/l	< 60
Alk. Phosphatase	138 U/l	40 – 129
CHE	5968 U/l	5320 – 12920
Bilirubin (total)	0.6 mg/dl	0 – 1,4
Bilirubin (direct / conjugated)	0.3 mg/dl	0 – 0,3
Bilirubin (indirect / unconjugated)	0.3 mg/dl	
Total protein	82.5 g/l	66 – 87
Albumin	43.8 g/l	35 – 52
Quick	>100 %	> 70
INR	0.98	0,85 – 1,15
PTT	34.6 sec.	25,9 – 36,6
Fibrinogen	392.9 mg/dl	210 – 400
Sirolimus	3.9	
Faktor II	92 %	70 – 120
Faktor V	123 %	70 – 120
Faktor VII	104 %	70 – 120
Faktor VIII	169 %	70 – 150
Protein C	74 %	70 – 140
freies Protein S (immunolog.)	96.2 %	66 – 149

C

Tumour markers	pre-therapeutic baseline	Range
AFP	731.0 ng/ml	< 7,0
CA 19-9	53.1 U/ml	< 27
CEA	2.2 ng/ml	< 3,8
PSA (total)	0.19 ng/ml	< 4,1
PSA (free)	0.07 ng/ml	
beta-HCG	0.63 mIU/ml	< 2,0

D

Infectiological parameters	pre-therapeutic baseline	Range
Anti-HAV-IgG	0.50 S/co	< 1,0
Anti-HAV-IgM	0.08 S/co	
HBsAg	0.20 S/co	
Anti-HBc	0.23 S/co	
Anti-HBs	0 IU/l	< 10
Anti-HCV	0.17 S/co	
Anti-HEV-IgG-ELISA	61.7 U/ml	0 – 24
Anti-HEV-IgM-ELISA	2.2 U/ml	0 – 24
Anti-HIV/p24-Antigen	0.13 S/co	< 1,0
TBC-Elispot	negative	
Anti-CMV-IgG (CMIA)	1.40 AU/ml	< 6
Anti-CMV-IgM (CMIA)	0.14 index	
CMV-DNA (PCR, stool)	0 copy/ml	
CMV-DNA (PCR, serum)	0 copy/ml	
Anti-Varizella Zoster-Virus, IgG	>2000 mIU/ml	
Anti-EBV-IgG-Immunoblot	positive	
Anti-EBV-IgM-Immunoblot	negative	

E

Autoimmune / rheumatologic parameters	pre-therapeutic baseline	Range
ANA (Titer)	1:320 Titre	< 1:40
autoimmunity against mitochondria	<1:40 Titre	< 1:40
AMA-M2	negative	
PCNA	negative	
Jo 1	negative	
Ro-52	negative	
SS-A	negative	
Sm	negative	
IgG	1809 mg/dl	700 – 1600
IgG-4	30.60 mg/dl	3,0 - 201
alpha1-Globulin	3.1 %	2,9 – 4,9
alpha2-Globulin	7.9 %	7,1 – 11,8
beta1-Globulin	4.3 %	4,7 – 7,2
beta2-Globulin	3.5 %	3,2 – 6,5
gamma-Globulin	16.8 %	11,1 – 18,8

Supplementary Figure 2: Overview of the routine clinical and laboratory examinations performed to exclude comorbidities such as secondary tumours, autoimmune diseases and occult infections.

A) Routine laboratory parameters
B) Biochemical graft liver function
C) Tumour markers to exclude secondary tumours
D) Infectiological parameters to exclude occult infections
E) Autoimmune / rheumatologic parameters

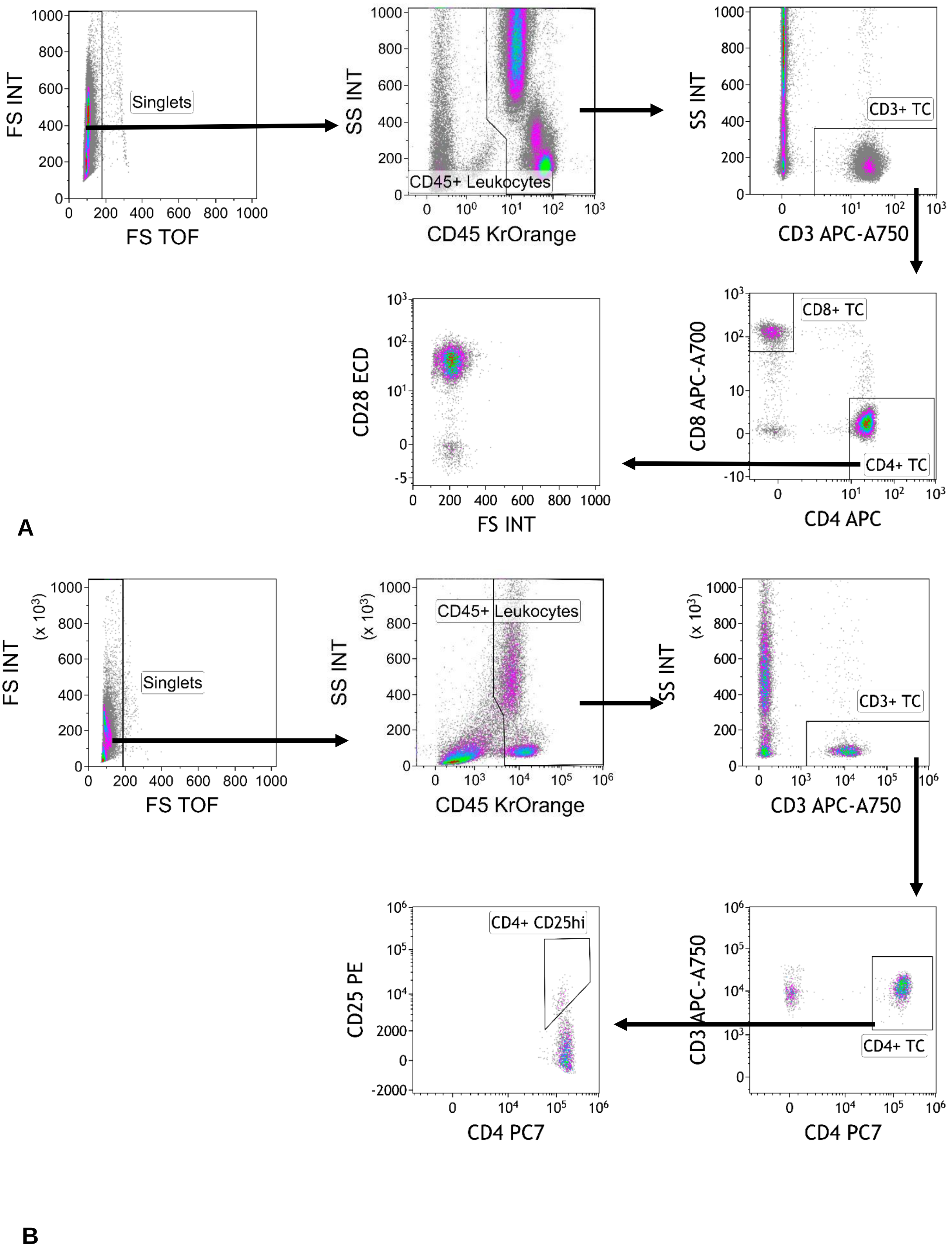

Supplementary Figure 3: Gating strategies used for immunomoditoring, as recommended by the manufacturer, to determine the CD28 expression level in CD4⁺ T cells (A) and the frequency of regulatory T cells (B).
